# Stereological investigation of the effect of *Elaeagnus angustifolia* fruit hydroalcoholic extract on osteoporosis in ovariectomized rats

**Published:** 2017

**Authors:** Mohmmad Hossein Dabbaghmanesh, Ali Noorafshan, Pedram Talezadeh, Nader Tanideh, Farhad Koohpeyma, Aida Iraji, Marzieh Bakhshayeshkaram, Nima Montazeri-Najafabady

**Affiliations:** 1 *Department of Endocrinology, Endocrinology and Metabolism Research Center, Shiraz University of Medical Sciences, Shiraz, Iran*; 2 *Department of Anatomy,* * Histomorphometry and Stereology Research Center, Shiraz University of Medical Sciences, Shiraz, Iran*; 3 *Department of pharmacology* *, Stem Cell and Transgenic Research Center, Shiraz University of Medical Sciences, Shiraz, Iran*; 4 *Researcher in Central Research Laboratory, Shiraz University of Medical Sciences, Shiraz, Iran*; 5 *Researcher* *in Health Policy Research Center, Shiraz University of Medical Sciences, Shiraz, Iran*

**Keywords:** Elaeagnus angustifolia, Stereology, Osteoporosis, Tibia bone, ALP, Rat

## Abstract

**Objective:**

Postmenopausal osteoporosis is characterized by increased fracture risk. However, each approved treatment has specific side effects. Therefore, foods with plant origins have increasingly attracted attention as an alternative treatment. Studies have shown that *Elaeagnus angustifolia* (EA) has antioxidant properties. The present study aimed to investigate the effects of EA hydroalcoholic extract on ovariectomy-induced osteoporosis in rats using stereological methods.

**Material and Methods:**

55 female Sprague-Dawley rats were randomly assigned to control, sham operated (normal saline), ovariectomized (OVX), OVX + EA fruit extract (600 mg/kg BW/day), and OVX + estradiol benzoate (3 mg/kg BW) for 16 weeks. Blood samples were collected to measure calcium, phosphorus, and alkaline phosphatase (ALP) plasma levels. Then, specimens from tibia and fifth lumbar vertebra (L5) bones were prepared and stereological analysis was done.

**Results:**

Ovariectomy significantly decreased the calcium level and increased the ALP level in the OVX group. In spite of improvement in calcium hemostasis in groups treated with estrogen and EA fruit extract (p<0.05), only treatment with estrogen was able to reduce ALP levels. Moreover, treatment with EA fruit extract and estrogen caused a significant increase in the number of osteoblasts in vertebra and tibia compared to the OVX group (p<0.05). Estrogen and EA fruit extract were also able to reduce the number of osteoclasts in tibia of the treated OVX rats (p<0.05).

**Conclusion:**

The results showed that EA extract exerted more effects, markedly, on osteoblastogenesis in the OVX rats. Thus, it could be considered as a potential agent to treat patients with osteoporosis.

## Introduction

Osteoporosis is characterized by low bone mineral density, structural deterioration of bone microarchitecture, and increased fracture risk (Jeddi et al., 2013 ▶). Hypoestrogenemia is an important cause of osteoporosis, which induces loss of bone mass in long bones and lumbar vertebrae (Gatta et al., 2015 ▶). Bone continually undergoes remodeling mediated by coordinated activities of bone resorbing cells (osteoclasts) and bone forming cells (osteoblasts). Loss of bone mass in osteoporosis is caused by an imbalance in bone remodeling, such a way that the rate of osteoclast-mediated bone resorption is higher than that of osteoblast-mediated bone formation (Bidwell et al. 2013 ▶,).

The majority of studies have shown oxidative stress as another important predisposing factor for osteoporosis (Manolagas, 2010 ▶). Estrogen deficiency lowers antioxidant levels and reduced antioxidant levels enhance bone loss (Sánchez-Rodríguez et al., 2007 ▶). In postmenopausal women, this acceleration of bone loss due to lack of estrogens can be associated with an increase in cytokine production by peripheral blood monocytes concomitant with generation of Reactive Oxygen Species (ROS) (Kim et al., 2012 ▶). 

Estrogens can also stimulate bone formation. Many studies have also indicated that Hormone Replacement Therapy (HRT) was associated with a reduction of hip, spine, and wrist fractures (Saleh et al., 2011 ▶). Although HRT has been proven efficacious in preventing bone loss, concerns about its side effects have led to interest in treatment modalities that not only decrease morbidity, but also have minimal side effects. Antioxidant nutrients might reduce the production of free radicals that contribute to bone resorption and enhance bone formation (Puel et al., 2004 ▶).

Herbs or medicinal plants have been used for treatment of numerous diseases for thousands of years. *Elaeagnus angustifolia *(EA), a plant native to western Asia, is a traditional herbal medicine. EA extract is rich in phytoestrogens and flavonoids that are known as natural estrogens and are capable to induce the transcriptional activity of the human estrogen receptor expressed in cultured cells by transient transfection (Bucur et al., 2008 ▶). Some studies have also shown anti-inflammatory effects of the fruits of this plant on rheumatoid arthritis and osteoarthritis (Nikniaz et al., 2014 ▶). This herbal medicine contains several chemical compounds, which usually exert their beneficial effects through multiple pathways and cover several targets. This property may help the treatment of the multifactorial pathogenesis of osteoporosis.

To date, evidence has demonstrated that a cocktail of different antioxidants might be more efficient compared to supplementation with a single active ingredient (Greenwald et al., 2007 ▶).

Since histomorphology of the bone may be altered in different sample preparation conditions, well-grounded stereological examinations provide quantitative morphological data on the most important characteristics. For the first time, the present study aims to quantitatively evaluate the effects of EA hydroalcoholic extract consumption on amelioration of ovariectomy-induced osteoporosis in rats. 

## Materials and Methods

**Drugs and chemical agents**

Estradiol benzoate was purchased from Jaber Ebne Hayan Pharmaceutical Company, Tehran, Iran. Ketamine 10% and Xylazine 2% were also purchased from Alfasan Company (Netherlands).

**Preparation of the hydroalcoholic extract**

EA fruits were purchased from Keshtvasanat Company, Beyza, Fars, Iran. Then, a plant voucher specimen (No. SoP UoG21001) was kept in the herbarium of Shiraz University. The pericarp was separated from the seed, dried in shadow, and grounded. After that, the pericarp powder (100 g) was added to 500 ml ethanol 70%. The obtained solution was left in the percolator at room temperature for 72 hr. Afterwards, the solvent was completely removed from the hydroalcoholic extracts by rotavapor at 40 ºC and dried in a vacuum desiccator (The efficiency of this method was 23.5%) (D’Amelio, 1999 ▶).

**Animal grouping and experimental design**

This work was approved by the Ethics Committee (No. 2225b125) of Shiraz University of Medical Sciences, Shiraz, Iran. In this study, 55 adult female Sprague-Dawley rats (12-14 weeks old and weighing 200±20 g) were purchased from the Laboratory Animal Center of Shiraz University of Medical Sciences. The rats were preserved under standard housing laboratory conditions (room temperature with relative humidity of 60±5%, temperature of 23±2 ºC, and 12 hr/12 hr light/dark cycles) and were fed with a standard pellet diet and water *ad libitum*. After one week of adaptation to the diet and the new environment, the animals were screened and those with body weight extremes were excluded from the study. Then, the rats were randomly divided into five groups each containing 11 animals. The animals in group 1 were considered as the control group, group 2 animals were sham operated, and those in groups 3 to 5 underwent ovariectomy. The rats in all the ovariectomized (OVX) groups were fed for 60 days to lose bone. Subsequently, the animals in groups 1, 2, and 3 received normal saline, those in group 4 were treated with oral EA fruit extract (600 mg/kg body weight daily), and group 5 received subcutaneous injection of estradiol benzoate (3 mg/kg body weight weekly) for four months. According to the results of other studies, the best suggested dose for EA fruit extract in regard with improving inflammatory condition was 600 mg/kg BW. Therefore, we decided to use this dose (Khodakarm-Tafti et al, 2015 ▶).

**Oophorectomy**

The adult female rats were bilaterally ovariectomized or sham-operated under anesthesia by ketamine 10% (100 mg/kg, Alfasan, Netherlands) and xylazine 2% (10 mg/kg, Alfasan, Netherlands). After ligation of the uterine horn through a midline longitudinal incision, both ovaries were surgically removed in all the groups, except for the control group. The sham-operated control rats had their ventral incision, but manipulation of their ovaries was performed without excising them.

**Collection of specimens and biochemical tests**

Eight and sixteen weeks after treatment with EA fruit extract, blood samples were collected in chilled non-heparinized tubes to clot at room temperature by cardiocentesis. The blood samples were centrifuged at 3500 rpm at 4˚C for 20 min and the separated sera were evaluated for biochemical markers, including calcium, phosphorus, and alkaline phosphatase (ALP). Serum creatinine and BUN levels were also quantified by calorimetric methods using kits supplied by Biosystems S.A, Spain. It should be mentioned that biochemical assessment was done 2, 4, and 6 months after the beginning of the study.

All rats were anesthetized with ketamine and xylazine solution intraperitoneally and sacrificed by using thiopental (100 mg/kg) at the end of the experiment. Then, each rat’s body weight and uterus weight were determined. Afterwards, the fifth lumbar vertebral body (L5) and the right tibia were isolated, cleaned of connective tissue, fixed in 10% buffered formalin, and decalcified in formic acid for stereological studies.

**Estimation of total phenolic compounds**

The Total Phenol Content (TPC) of the plant extract was evaluated using the modified Folin-Ciocalteu spectrophotometric method described previously by Waterhouse (2002). ▶ This method involves reduction of Folin-Ciocalteu reagent by phenolic compounds, with a concomitant formation of a blue complex (Waterhouse, 2002 ▶). In this study, an aliquot of 40 µL solution of the extract was mixed with 3.16 ml water and 200 µL Folin-Ciocalteu reagents. The mixture was incubated for 8 min. Then, 600 µl of 0.25% sodium carbonate was added to this solution. The obtained solution was further incubated at room temperature for 2 hr and its absorbance was measured at 765 nm against the blank sample. The same procedure was repeated for gallic acid standard solution, and the calibration standard curve was construed. The measurement was then compared with the standard curve prepared for gallic acid solution. The concentration of the total phenol was expressed as milligrams of Gallic Acid Equivalents (GAE) per gram of dry extract (mg of GA/g of dE) (Noorafshan et al., 2015 ▶). Based on the calibration curve’s equation, the TPC in 80% ethanol extract was 8.75 mg GAE/g dry extract.

**Estimation of bone volume**

**Figure 1:**
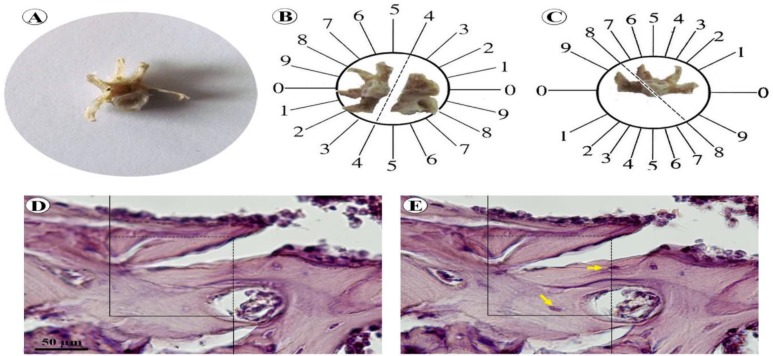
The stereological methods. A lumbar vertebra was dissected out (A). Orientator method was used to obtain isotropic uniform random sections of the bone (B, C).Optical dissector method (D). An unbiased counting frame was lied on the images (E). The cell nuclei, which were not in contact with the forbidden lines (bold lines), were counted (arrows

Sherle’s method was used to estimate the bones’ volume (Karbalay-Doust et al., 2012 ▶). Subsequently, the vertebra and tibia were decalcified and processed histologically. The orientator technique was applied to obtain the isotropic random sections of the bones (Mattfeldt et al., 1990 ▶) (Figures 1 A, B and C). Briefly, the bones were placed on two circles (an equidistance divided circle and a non-equidistance divided circle) and were sectioned using a blade. In this way, 10-12 slabs were sampled from each bone. An aspherical piece of about 1 mm diameter was punched out from a random slab using a trocar. Thereafter, the diameter and area of the circular pieces were calculated. The sampled slabs and the circular pieces of each animal’s bone were embedded in one paraffin block. Then, 5 and 25 µm sections were obtained. After staining the tissue sections with hematoxylin and eosin, the area of the circular pieces were measured again to approximate the global degree of bone tissue shrinkage. These methods allowed us to estimate the Degree of shrinkage (Dsh), which could be induced by tissue processing and staining procedures. Finally, the following formula was used (Nyengaard, 1999 ▶; Gundersen et al., 1988 ▶):

$\mathrm{Degree of shrinkage}=1-{\left(\frac{\mathrm{Area after}}{\mathrm{Area before}}\right)}^{1.5}$

where “area after” and “area before” were the areas of each circular piece of the bones after and before processing and staining, respectively.

The final volume of the bone was also calculated using the below formula:

V_ (final)_ = (1-Dsh) × V_ (primary)_

**Figure 2:**
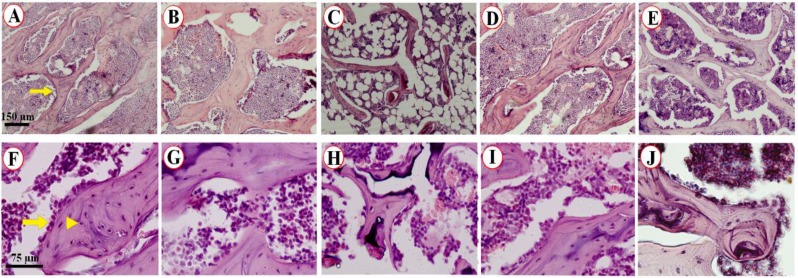
The qualitative microscopic evaluation of the L5. The trabeculae showed normal appearance in the control (A) and sham-operated (B) rats. The trabecular atrophic changes could be seen obviously in the OVX rats (C). Treatment of the OVX animals with estrogen (D) and Elaeagnus angustifolia (E) showed protective changes in the bone. Survey of the number of osteoblasts and osteoclasts indicated a normal population in the control (F) and sham-operated (G) animals. A massive cell reduction could be observed in the OVX animals (H). The osteoblasts and osteocytes were protected in the animals treated with estrogen (I) and Elaeagnus angustifolia (J). The scale bar was 150 µm for the upper row Figures and 75 µm for the lower row. The arrow in A indicates bone trabeculae. The arrow and arrowhead in F indicate the osteoblast and osteocyte, respectively

**Estimation of the volume density of the bones’ trabeculae**

The volume density of the trabeculae “Vv (trabeculae)” refers to the proportion of the unit volume of the osseous tissue that is filled by trabeculae. Briefly, volume density of the trabeculae was evaluated on 4 µm thickness sections through the point-counting method and using Delesse’s formula (Karbalay-Doust et al., 2012 ▶, Nyengaard, 1999 ▶):

$V_{V}(\mathrm{trabeculae}/\mathrm{bone})=\frac{P(\mathrm{Trabeculae})}{P\left(\mathrm{reference}\right)}$

Where “P (trabeculae)” was the number of the test points falling on the trabeculae and “P (reference)” was the number of the points falling on the reference tissues (trabeculae plus cavities) of L5 and tibia bones. The following formula was used to estimate the absolute trabecular volume (Noorafshan et al., 2012 ▶):

V (bone) = V (final) × V_v _(bone)

**Estimation of the numerical density and absolute number of bone cells**

Numerical density “NV (cells/bone)” refers to the number of cells per unit volume of the trabeculae and was evaluated using Disector method (Figures 1 D and E). Briefly, a 25-µm section was evaluated by moving down through the z-axis optical plane of the tissue using a microscope (Nikon E-200, Japan) linked to a monitor. An unbiased counting frame was lied on the tissue’s live image to sample the bone cells (Noorafshan et al., 2012 ▶). Upper and lower guard zones of the osseous tissue were set to be 5 µm using a microcator (MT 12, Heidenhain, Germany). The average final section thickness “t” was also measured on the tissue using the microcator (20 µm). Besides, an optical section (10 µm) was considered as the disector’s height. The numerical density of the cells was measured using the following formula:

Nv(cells/bone)=∑i=1nQ(∑P×h× af)×tBA

Where “ΣQ” was the number of the cells counted in all the Disectors, “h” was the height of the optical Disector, “a/f” was the area of the counting frame, “ΣP” was the total number of the counted frames, “BA” or block advance was the setting of the microtome to cut the paraffin block, and “t” was the mean of the final section thickness. 

To estimate the total number of the bone cells, the following formula was applied:

N (bone cells) =N_V_ (cells/bone) ×V (final)

**Statistical analysis**

The SPSS statistical software (v. 18) was used to carry out statistical analysis. First, normal distribution of the data was assessed. Then, the data were analyzed by Mann-Whitney U test. The results were presented as mean ± standard deviation, and significance level was set as p≤0.05.

## Results

**Body weight and uterus weight**

The mean body weight was 225±32 g in all study groups at the beginning of the experiment. After 6 months, all rats were weighed again and no noticeable increase was observed in their body weight. However, the weight of the uterus significantly decreased in the OVX rats compared to those with intact ovaries (p=0.001).

**Biochemical parameters**

Serum calcium, phosphorus, and ALP levels in the experimental groups are shown in Table 1. As the Table depicts, no significant difference was found among the experimental groups regarding serum calcium levels at the beginning of the experiment. However, OVX caused a significant decrease in the calcium levels in the untreated OVX group compared to the sham, control, and EA fruit extract-treated groups at the end of the study (p=0.001). Nonetheless, no significant change in serum phosphorus level was observed in the control, sham, and extract-treated animals at all stages of the study.

The results showed a significant increase in plasma ALP level in the OVX rats compared to the sham-operated and control groups in the first stage of the study (p=0.001). Yet, administration of estradiol resulted in a decrease in bone turnover marker during the treatment. Nevertheless, no significant difference was seen between the estrogen-treated rats and the sham-operated and control groups with respect to ALP levels. In spite of improvement in calcium hemostasis, no significant decrease in bone turnover marker (ALP) was observed in the EA fruit extract-treated group compared with the estrogen-treated group. In addition, no significant differences were found between the EA fruit extract-treated rats and control and sham groups in this regard. Furthermore, no significant change was found in serum creatinine and BUN levels in any of the experimental groups before and after the treatment.

**Table 1:** Biochemical parameters in the experimental and control groups

**Blood parameters**	**Time** **(month)**	**Control**	**Sham**	**OVX**	**OVX + EA**	**OVX + estrogen**
**Ca (mg/dl)**	2 4 6	9.7 ± 0.5 9.8 ± 0.8*† 10.2 ± 0.5*	9.6 ± 0.25 10.1 ± 0.3*† 9.6 ± 0.6*	9.8±0.3 8.1±1.0 8.2±0.6	9.6±0.2 8.9±0.7 9.1±0.2*	9.5±0.2 9.8±1*† 10.9±0.6*‡
**P (mg/dl)**	2 4 6	5.1 ± 1.0 5.9 ± 0.8 6.6 ± 1.9	4.6 ± 0.8 5 ± 0.8 6.6 ± 0.8	4.2 ± 0.70 6.5 ± 1.0 5.2 ± 0.8	4.5±0.9 6.7±0.2 6.6±0.8	4.1±0.4 6.7±.6 6.6±0.8
**ALP (mg/dl)**	2 4 6	333± 143*†‡ 311± 151*† 324± 146*†	391 ± 94*†‡ 313 ± 124*† 295 ± 144*†	465±85 410 ± 82 466± 101	504±100 433±102 511±83	518±132 265±110† 379±118†

CA: Calcium, P: Phosphorus, ALP: Alkaline Phosphatase

† p< 0.05, OVX + EA vs. control or sham-operated groups or estrogen.

‡ p<0.05, control or sham vs. estrogen.

* P<0.05, OVX vs. control or sham or estrogen or EA extract groups

**Stereological study**

The weight of the vertebrae and tibia, volume of the vertebrae and tibia, total volume of the bones’ trabeculae, and total number of the osteocytes, osteoblasts, and osteoclasts are presented in Tables 2 and 3. Additionally, the qualitative microscopic evaluation of the L5 was shown in Figure 2.

**The weight of the vertebrae and tibia**

According to Table 2, the weight of L5 significantly decreased by 31% in the OVX group in comparison to the control and sham-operated groups (p<0.05). 

However, the weight of L5 increased in the EA fruit extract- and estrogen-treated groups compared to the OVX groups, but the difference was not statistically significant.

Moreover, the weight of the tibia was significantly lower in the OVX group compared to the sham and control groups (p<0.05) (Table 3). Besides, the mean weight of the tibia significantly increased in both OVX groups receiving EA fruit extract and estrogen supplement compared to the OVX group (p<0.05). Nevertheless, no significant differences were found among the control, sham-operated, and treated groups regarding the weight of the vertebrae and tibia.

**Table 2:** Stereological parameters of vertebra in control, sham-operated, and ovariectomized (OVX) rats with or without treatment with Elaeagnus angustifolia extract (600 mg/kg/day) and estrogen (3 mg/kg/week

	**Weight (mg)**	**Volume (mm** ^3^ **)**		**Number (×10** ^6^ **)**		
**Groups**	**Vertebra**	**Vertebra**	**Trabeculae**	**Osteocytes**	**Osteoblasts**	** Osteoclasts**
**Control**	272±23	180±35	118±20	1.67±0.40	0.60±0.27	8.0±4.8
**Sham-operated**	292±45	179±17	120±21	1.84±0.46	0.61±0.09	7.4±2.7
**OVX**	201±10 †	125±6 †	67±12†	0.98±0.17 †	0.19±0.10†	23.6±11†
**OVX+estrogen**	248±22	199±9*	82±10	1.61±0.54	0.61±0.21 *	17.7±9.4
**OVX+ EA**	245±30	196±15*	72±6	1.23±0.37	0.63±0.21 *	18.04±8.3

EA: *Elaeagnus angustifolia*.

† p< 0.05, OVX vs. control or sham-operated groups.

* p<0.05, OVX vs. OVX+ estrogen or EA extract groups.

**Table 3:** Stereological parameters of tibia in the control, sham-operated, and ovariectomized (OVX) rats with or without treatment with Elaeagnus angustifolia extract (600 mg/kg/day) and estrogen (3mg/kg/week

	**Weight (mg)**	**Volume (mm** ^3^ **)**		**Number (×10** ^6^ **)**		
**Groups**	**Tibia**	**Tibia**	**Trabeculae**	**Osteocytes**	**Osteoblasts**	**Osteoclasts**
**Control**	372±31	233±20	158±31	26.55±7.93	5.15±2.85	90.5±7.8
**Sham-operated**	364±41	210±20	156±31	23.32±3.57	5.33±1.54	83.5±12.3
**OVX**	219±35†	139±36 †	86±16 †	11.20±4.82 †	1.27±0.61 †	399.7±106.2 †
**OVX+estrogen(3mg/kg/week)**	372±61*	243±32*	110±22	27.62±11.95*	5.51±4.47*	154.6±100.4*
**OVX+EA(600mg/kg)**	376±57*	236±17*	99±9	20.29±3.73	7.86±2.56*	255.1±154*#

† p< 0.05, OVX vs. control or sham-operated groups.

* p<0.05, OVX vs. OVX+ estrogen or EA extract groups.

# p<0.05, OVX+ EA extract vs. control or sham groups

**The volume of the vertebrae and tibia**

On the average, the volume of L5 and tibia decreased by 30% in the OVX group in comparison to the sham and control groups (p<0.05). However, treatment of the OVX rats with 600 mg/kg EA fruit extract and estrogen significantly increased the mean volume of L5 and tibia (p<0.05). Nonetheless, no statistically significant difference was observed among the control, sham, estrogen, and EA fruit extract-treated rats regarding the volume of the vertebrae and tibia. 

**Total volume of the bones’ trabeculae**

The volume of the trabeculae of L5 and tibia decreased by averagely 44% in the OVX group compared to the sham and control groups (p<0.05). Nonetheless, no significant differences were observed between the OVX and EA fruit extract-treated OVX groups in this regard.

**Total number of osteocytes**

The results of our study showed a 41% decline in the total number of osteocytes in the total volume of the L5 trabeculae in the OVX group compared to the control and sham-operated groups (p<0.05). However, an increase was observed in the total number of osteocytes in the EA fruit extract- and estrogen-treated groups in comparison to the OVX group, but the difference was not statistically significant. On the other hand, no significant difference was found among the OVX + EA fruit extract-treated, estrogen-treated, sham, and control groups concerning the number of osteocytes.

In the tibia, the total number of osteocytes was 57% and 51% lower in the OVX group compared to the control and sham-operated animals, respectively (both p<0.05). Nonetheless, an increase was observed in the total number of osteocytes in the EA fruit extract- and estrogen-treated groups in comparison to the OVX group, but the difference was only significant in the group receiving estrogen (p=0.002). Nevertheless, no significant difference was found among the EA fruit extract-treated, control, and sham-operated groups with respect to the number of osteocytes.

**Total number of osteoblasts**

In the tibia, the total number of osteoblasts was 57% and 51% lower in the OVX group compared to the control and sham-operated animals, respectively. The number of osteoblasts in the L5 and tibia were also 70% and 75% lower in the OVX group compared to the control and sham groups, respectively (p<0.05 for both). However, a significant increase in the number of osteoblasts was seen in the EA fruit extract- and estrogen-treated groups compared to the OVX group (p<0.05). Yet, no significant difference was seen among the EA fruit extract-treated, control, and sham-operated groups in this regard.

**Total number of osteoclasts**

The number of osteoclasts in the L5 was 3-fold higher in the OVX rats compared to the sham and control groups (p<0.05 for both). However, the number of osteoclasts decreased in the L5 of the EA fruit extract- and estrogen-treated groups compared to the OVX groups although the difference was not statistically significant. In addition, no significant differences was found among the control, sham-operated, and treated groups regarding the total number of osteoclasts.

Furthermore, the number of osteoclasts in the tibia significantly increased by 4.5 folds in the OVX rats compared to the sham and control groups. On the other hand, the number of osteoclasts significantly decreased in the EA fruit extract- and estrogen-treated groups compared to the OVX group (p<0.05 for both). Additionally, the results showed a significant difference between the EA fruit extract-treated, but not estrogen-treated, and control and sham groups in this regard.

## Discussion

In osteoporosis, an imbalance between bone resorption and formation is due to extension of lifespan of osteoclasts and shortening of lifespan of osteoblasts and possibly intestinal absorption of calcium is impaired (Ashouri et al., 2015 ▶). Estrogen deficiency-induced bone loss is a complex interaction of estrogen, the immune system, and the bones. Estrogen maintains bone homeostasis through regulatory effects on the immune system and oxidative stress and direct effects on bone cells (Noorafshan et al., 2015 ▶). Treatment of postmenopausal osteoporosis involves use of drugs and hormone therapy, but each approved treatment has specific side effects. In general, two types of drugs are used in treatment of osteoporosis, antiresorptive and anabolic agents. Most drugs, such as estrogen, are antiresorptive agents (Choi, 2015 ▶). As an alternative treatment, foods with plant origins attracted increased attention because of their potential benefits and reduced adverse effects. It is noticeable that many plants have the potential to prevent and treat osteoporosis. However, only a restricted number of these plants have been meticulously investigated up to now (Jia et al., 2012 ▶). EA, a commonly prescribed herb in Western Asia for management of bone diseases (Ebrahimi et al., 2014 ▶; Zargari, 1990 ▶), is known for its effects on strengthening the bone. It was demonstrated that peel, fruit, and seed of this fruit contains different flavonoids, polyphenols, and terpenoids (Farzaei et al., 2015 ▶).

In the present study, OVX led to a significant decrease in calcium concentration compared to the other groups, but serum levels of phosphorus remained unchanged. Finally, however, no significant change was observed among the control, sham, estrogen-treated, and EA extract-treated animals regarding serum calcium level. Menopause is linked to decreased intestinal calcium absorption and increased renal excretion of calcium (Hohman et al., 2015 ▶). Calcium deficiency leads to worsening of bone deposition. Some studies demonstrated that phenol compounds in plants were able to enhance intestinal absorption of calcium, deposition of calcium ions in osteoblastic MC3T3-E1 cells, and inhibition of osteoclast formation (García-Villalba et al., 2014 ▶).

Bone is metabolically active and is continually repaired and remodeled throughout an individual’s life. Bone cells activity can be evaluated through biochemical markers. Most biochemical indices of bone resorption are related to collagen breakdown products or osteoclast-specific enzymes. Markers of bone formation are either by-products of collagen neosynthesis or osteoblast-related proteins, such as ALP (Garnero, 2014 ▶). Generally, all bone biochemical markers markedly increase at the beginning of menopause. Bone loss is accompanied by a significant increase in bone remodeling, as evidenced by increased biochemical bone turnover markers, such as serum ALP (Mukaiyama et al., 2015 ▶). ALP plasma concentration is an indicator of bone formation and a major index of osteoblastic activity (Xufeng et al. 2014 ▶). In the current study, plasma ALP concentration significantly increased in the OVX rats in comparison to the sham-operated and control groups. Administration of estrogen significantly reduced bone turnover, which was accompanied by a decrease in serum ALP level. However, at the end of the study, the results indicated that ALP level was significantly higher in the *EA*-treated animals compared to the control, sham, and estrogen-treated groups. EA contains phenolic compounds, based on an *in vitro* study, a mixture of phenolic acids significantly upregulated ALP gene expression and stimulated osteoblast differentiation, resulting in significantly increased bone mass (Chen et al., 2010 ▶).

Based on the literature, this was the first study investigating the anti-osteoporotic effects of EA stereologically. Assessment of tibia and L5 showed that OVX rats had a lower total volume of the bone trabecular and higher trabecular separation compared to the other groups, indicating increased bone fragility (Peel, 2009 ▶). In agreement with the previous studies, the increased number of osteoclasts and decreased number of osteoblasts and osteocytes in the OVX rats pointed toward increased bone resorption, while these changes were modified in EA*-* and estrogen-treated groups (Nishide et al. 2013 ▶). In our study, administration of estrogen or EA to osteoporotic rats decreased the number of osteoclasts and significantly increased the number of osteoblasts. After the onset of menopause, drop in the blood level of estrogen results in bone loss and increases the incidence of osteoporosis (Khosla et al., 2012 ▶). Many studies have acknowledged the role of pro-inflammatory cytokines in the etiology and pathogenesis of osteoporosis. Some evidence has also linked bone loss to ROS. Estrogen deficiency provokes oxidative stress, impairs bone antioxidant defense, increases lipid peroxidation and H_2_O_2_ and diminishes enzymatic antioxidants, such as super oxygen dehydrogenase and glutathione peroxidase (Goldring et al., 2015 ▶). Estrogen deficiency also upregulates the formation of osteoclasts and osteoblasts by induction of the production and activity of cytokines, including IL-6, TNF, IL-1, and Macrophage Colony Stimulating Factors (M-CSF) (Callaway et al., 2015 ▶). Two phytosterols have been detected in EA*, *namelyβ-sitosterol and stigmasterol (Si et al., 2011 ▶, Bekker et al., 1997 ▶). Phytosterols, which have estrogen-like activity, enhance differentiation and proliferation of primary osteoblasts by increasing the mRNA expression of ALP and might modulate osteoclastogenesis via regulation of Osteoprotegerin (OPG) and RANKL mRNA expression in bone cells (Mok et al., 2010 ▶).

It has also been shown that EA has anti-inflammatory effects in animal models (Nikniaz et al., 2014 ▶). Besides, ethanolic extract of *EA* possesses antioxidant activity (Chen et al., 2014 ▶, Wang et al., 2013 ▶). The antioxidant activity of this extracts was linearly related to polyphenols, but non-linearly related to flavonoids (Bucur et al., 2008 ▶).

In contrast to the effect of other antiremodeling plant extracts on bone that mainly modulate and inhibit osteoclastogenesis demonstrated with decrease in the bone turnover markers (decrease in serum ALP levels compared to the OVX group) (Noorafshan et al., 2015 ▶), the current study findings demonstrated that *EA* exerted an uncoupling bone formation with a significant increase in osteoblasts count in the *EA* extract-treated group compared to the OVX group, which was shown biochemically with increased ALP levels in the *EA* extract-treated group. 

Recent studies have suggested that postmenopausal osteoporosis might be due not only to augmented osteoclast formation and to activity, but also to an increase in osteoblastic inhibition and a decrease in osteoblastic activity (D’Amelio et al., 2011 ▶). Over the last years, anabolic treatment has been anticipated as the therapy for postmenopausal osteoporosis. These drugs significantly diminish the risk of vertebral and non-vertebral fragility fractures (Greenspan et al., 2007 ▶). Although suppressed osteoclastogenesis might be considered in determining increased bone mass in *EA*-fed animals, increased bone mass in these animals was associated with increased ALP level, osteoblast number, bone mineralization, and bone volume. 

This is the first stereological and a preliminary study to evaluate the possible use of EA in treatment of osteoporosis. However, considering the results of this study, future research should focus on isolated or mixture of active constituents to determine the mechanisms underlying the bone effects and to reveal the beneficial therapeutic and safety properties of its phytochemicals, as a complementary and alternative medicine for management of osteoporosis.

The results of this study provided a basis for clinical evaluation and demonstrated the potential effects of EA extract, as a herbal drug. The findings suggested that EA extract could be an effective natural anabolic alternative for treatment of postmenopausal osteoporosis. Yet, further research are needed to be conducted in this area.
